# Factors Associated with Hypertension Care Follow-Up in the Ethiopia HEARTS Program

**DOI:** 10.5334/gh.1407

**Published:** 2025-02-26

**Authors:** Girma A. Dessie, Meron H. Beyene, Manuel K. Sibhatu, Bolanle Bangibe, Bishal Belbase, Dalya Samarah, Henok G. Kebede, Arone H. Mebrhatu, Hailemichael Getachew, Endawoke A. Alemayue, Addisu Worku, Andrew E. Moran

**Affiliations:** 1 Resolve to Save Lives Country office Addis Ababa, Ethiopia; 2 Resolve to Save Lives Global office New York, USA; 3 Armauer Hansen Research Institute, Ethiopia; 4 Federal Ministry of Health, Ethiopia; 5 Columbia University Irving Medical Centre, USA

**Keywords:** Hypertension, World Health Organization, HEARTS, health services, Ethiopia, low- and middle-income countries

About 11.8 million Ethiopian adults (16%) are living with hypertension, and among them less than 2% have their blood pressure (BP) controlled (<140/90 mmHg) (1). Controlling hypertension is a public health priority for Ethiopia, where 16% of deaths are due to cardiovascular diseases (2). In 2020, Resolve to Save Lives, the World Health Organization (WHO) and Ethiopia’s Federal Ministry of Health launched the Ethiopia HEARTS hypertension control program in 68 primary health care units (PHCs); 58 health centres and 10 primary hospitals located in seven regions of Ethiopia (3). The Ethiopia HEARTS program implemented the five components of the WHO-HEARTS hypertension control technical package: a simple treatment protocol with defined drugs and doses; reliable supply of antihypertensive medicines and BP monitoring devices; team-based care including non-physician health care workers; community-based care; and a robust health information system (digital Simple app hypertension management tool) (4–5). Patients enrolled in the Ethiopia HEARTS program were expected to return monthly to the PHCs for hypertension care. By July 2022, Ethiopia HEARTS had enrolled 12,863 patients on hypertension treatment, of whom 45% had controlled BP. While this represented an improvement from the baseline controlled BP of 22%, loss to follow-up impeded progress towards furthering growth of the program and improvement of hypertension control. At that time, 33% of patients did not return for clinic follow-up visits in the prior three months, and 12% had not visited in the prior year. A survey was conducted to identify factors associated with failure of Ethiopian people with hypertension to follow up with scheduled HEARTS hypertension management visits.

Between October 11–30, 2021, concurrent with the COVID-19 pandemic, a cross-sectional community-based survey was conducted on consecutive adults aged ≥ 18 years with hypertension diagnosed before July 2021, registered in the Ethiopia HEARTS hypertension control program, reported to their local PHC, and provided informed consent. The study sample comprised the Ethiopian rural population seeking medical care in government-run public sector primary health care facilities. Trained enumerators approached eligible patients with hypertension visiting the facility, briefed them about the survey and invited potential participants to participate in the survey. Those expressing interest and providing written informed consent were enrolled. Participant responses were entered into an encrypted digital device. Details on the design of the survey, statistical analysis, survey questionnaire, and script for soliciting informed consent are provided in **Online Appendices A–C**. The survey obtained participant information on:

1. Identifying and demographic information (age, sex, education and income level, travel time to PHC).
2. Access to health services and hypertension services financing information.
3. Reasons for missed or delayed hypertension services.
4. Continuity of hypertension services, including access to blood pressure measurement and antihypertensive medicines.

The survey protocol was approved by the Armauer Hansen Research Institute Ethics Review Committee in Addis Ababa and the Biomedical Research Alliance of New York Institutional Review Board in the USA. Factors associated with individual patient access to hypertension services were summarised as counts and proportions. The proportion of participants missing a scheduled hypertension management visit in the prior three months was calculated. Logistic regression models all used missed visits in the prior three months as the dependent variable, and health services access factors were entered one at a time as independent variables. These included travel time to reach a PHC, payment method, and appointment spacing. Each of these individual models was adjusted for survey participant age, sex, income, education level and residence (urban, semi-urban, or rural).

Around 1,240 patients seeking care at the study’s PHCs were randomly selected and approached. Of these, 1,156 (93%) agreed to participate in the survey, provided informed consent, and were enrolled. The mean age of the study participants was 58.7 years (standard deviation: 12.5) with an age range from 20 to 96 years. Most participants (59.4%) were female. The majority of participants (61.2%) reached their PHC by foot; another 16.9% used public transportation (Table 1). About 13.6% of participants lived 31–60 minutes from the PHC and another 4.15% lived more than an hour away. About half of the patients received hypertension services covered by government health insurance coverage; the remaining half paid out-of-pocket.

**Table 1:** Baseline Health Service access of survey participants, all who were enrolled in the Ethiopia HEARTS hypertension control program, October 2021 (n = 1156).


VARIABLES		RESULTS	

		COUNT	%

**Socio-demographic factors**			

**Means of transportation to primary health care center (PHC)**	Bicycle	6	0.5

	Carpooling horse cart	11	0.9

	Motorcar motorcycle or scooter	129	11.2

	Multiple means	105	9.0

	Public transportation	195	16.9

	Walking	707	61.2

	Other	3	0.3

	**Total**	**1156**	**100**

**Time to reach PHC**	10 minutes	320	27.7

	11 to 30 minutes	631	54.6

	31 to 60 minutes	157	13.6

	> 60 minutes	48	4.2

	**Total**	**1156**	**100**

**Health system factors**			

**Time required for typical PHC visit for HTN care**	1 hour	470	40.7

	1 to 3 hours	601	52.0

	half days	83	7.2

	full days	2	0.2

	**Total**	**1156**	**100**

**HTN services payment Method**	Services fee waiver	52	4.5

	Insurance coverage	596	51.6

	out of pocket	502	43.4

	Other	6	0.5

	**Total**	**1156**	**100**

**Appointment frequency (visit spacing)**	Every month	849	73.4

	Every two months	226	19.6

	Every three months	75	6.5

	Other	6	0.5

	**Total**	**1156**	**100**


About 17.8% of hypertensive patients enrolled in the Ethiopia HEARTS program reported missing their scheduled hypertension management visits in the prior three months. Through logistic regression analyses, it was found that patients paying for hypertension services out-of-pocket were 1.6 times more likely to miss scheduled hypertension care than those who had insurance or other free services access [95% confidence interval (CI) 1.2–2.2; p-value 0.003). Patients living with a travel time of more than one hour from their PHC were 3.1 times more likely to miss their appointment compared with patients living one hour away or less [95% CI 1.6–6.2; p-value 0.001]. Lastly, patients with monthly appointments were 2.6 times more likely to miss their appointment compared with patients with appointments two and more months apart [95% CI 1.1–6.2; p-value 0.028].

High rate of loss to follow-up acts as a hinderance on achieving the program’s hypertension control goals as missed follow-up visits and medication prescriptions can put patients at higher risk of cardiovascular disease. Understanding reasons for patients’ poor follow-up can guide the design and adoption of countermeasures to improve retention in care. This cross-sectional survey of Ethiopian people with hypertension enrolled in a HEARTS hypertension control program identified three factors associated with missed hypertension care visits: high out-of-pocket payments for hypertension services, a travel time of ≥ 60 minutes to a PHC, and frequent, monthly hypertension management visits.

Based on the results of this survey, Ethiopia HEARTS introduced pilot interventions to improve patient retention at the beginning of 2022. Interventions included a three-month visit spacing and prescription refills for stably controlled people with hypertension. Currently, nearly one in five HEARTS patients receives multi-month drug refills. Simultaneously, the government has already expanded coverage of community-based health insurance (CBHI), which ensured zero out-of-pocket medication costs for non-communicable disease patients. According to a separate 2022 survey of the HEARTS PHCs, an estimated 60% of the catchment population subscribed to CBHI, with coverage progressively increasing over time. These combined efforts significantly improved retention of hypertension (HTN) patients in care from 52% in June 2021 to 76% By December 2022, Ethiopia HEARTS improved the BP control rate to 57%. Based on the results of this study, we recommend that the government of Ethiopia address the barrier of distance from PHCs by providing some services for stably controlled patients (e.g., prescription refills) at sub-centres nearer to the patients’ homes.

## Additional Files

The additional files for this article can be found as follows:

10.5334/gh.1407.s1 Supplementary Material. Appendix A.

10.5334/gh.1407.s2 Supplementary Material. Appendix B.

10.5334/gh.1407.s3 Supplementary Material. Appendix C.
