# Supplementary material for: Factors Associated with Hypertension Care Follow-Up in the Ethiopia HEARTS Program
Source: Glob Heart. 2025 Feb 26;20(1):20. doi: 10.5334/gh.1407 (PMC11869820; doi:10.5334/gh.1407)
Supplement: Supplementary Material. — Appendix A. [file gh-20-1-1407-s1.pdf]

Supplementary Appendix A

Survey Protocol: Factors Affecting Hypertensive Care Follow Up in Ethiopia

October 2021

Addis Ababa, Ethiopia

Acknowledgments

Resolve to Save Lives (RTSL) would like to thankfully acknowledge the contributions of everyone involved in the design and data collection of this study. Each person's contributions were vital in making the study a success.

Our gratitude is directed in particular to:

- The research team who are leading the study in every step and finalize this important study.
- The global Resolve to Save Lives team who unreservedly support the survey from inception to very end technically.
- To Armauer Hansen Research Institute (AHRI) for their technical support in designing of this study.
- To Ethiopia Federal Ministry of Health for their contribution in the data collection process and for granting access to their server.
- To all data collectors, supervisors, and health care facility staff who engaged in the data collection process.
- Last, but not least, to all study participants who are willing to give their time to be interviewed.

Acronyms

AHRI	Armauer Hansen Research Institute
CBHI	Community Based Health Insurance
CHEWs	Community Health Extension Works
DSD	Differential Service Delivery
EHCI	Ethiopia Hypertension Control Initiative
HTN	Hypertension
ICC	Intra-cluster correlation coefficient
MOH	Ministry of Health
NCD	Non-Communicable Disease
PERC	Property and Environment Research
PHC	Primary Health Care
PI	Principal Investigator
RTSL	Resolve To Save Lives
WHO	World Health Organization

Methods

Survey Design

A self-report survey was designed to assess Hypertension Services access for patients participating in the Ethiopia HEARTS program after the program in October, 2021, after the program had been active for about one year. The survey was orginally designed to be a “before and after” repeated survey and powered to detect differences between intervention and control facilities; however, due to the onset of civil war and resulting disruptions in primary health care, the planned follow up survey was cancelled. This report therefore was limited to the baseline survey only.

Study Area and Participants

All hypertensive patients enrolled to care and treatment and included in the official patient register at the primary health care facilities in the five regions (Addis Ababa, Amhara, Dire Dawa, Oromia and Sidama) which are supported by EHCI were eligible. Specifically, adults (age ≥18 years) with a diagnosis of hypertension recorded before July 1, 2020 and living within the woredas were eligible for the survey. Hypertension was diagnosed when the patient had a seated systolic BP >=140 mmHg or diastolic BP >= 90 mmHg measured with a validated oscillometric devices by trained nurses or medical officers using standard technique on two separate occasions. Potential participants were excluded if they carried a diagnosis of COVID-19 within 14 days of the planned enrollment interview, they were pregnant, or they were living with a terminal medical condition.

Participants were sampled consecutively upon presentation for a routine clinic visit to two types of primary health care facilities. First, 32 primary health care clinic (PHC) sites were sampled from government primary health care facilities implementing the WHO-HEARTS technical package. These PHCs had already received 12 months of program support, including a start-up supply of medicines and blood pressure monitoring devices, training for health care workers, regular mentoring and program monitoring. The HEARTS sites were intended to be sites that would receive a package of quality improvement interventions. A second set of 32 usual care health PHC facilities were originally intended to serve as a control site/group.

Eligible hypertension patients arriving to the primary health care facility were approached by study staff, who were trained enumerators. Enumerators described the brief community-based survey and invite potential participants to take the survey. People expressing interest in providing responses to the survey were enrolled, after obtaining their written informed consent.

Sample Size

The sample size was calculated based on the assumption of p-value of a baseline prevalence of missed essential medical services of <22% (based on a prior survey of missed PHC visit prevalence among the general Ethiopian adult population during Covid-19 epidemic service disruptions but for a longer look-back period of "since the COVID-19 crisis"). Expected differences in intervention versus control missed visit patient proportion at 12 months (the timing of the follow up survey) was 10 percentage points, the alpha level (type 1 error) of 0.05, and power of 0.9 and a 1:1 ratio between the control and intervention. The survey was administered in 32 PHCs in EHCI/intervention and 32 PHCs.

Patient selection at PHCs was conducted using a standard primary health care clinic registration book for hypertension and diabetes as well as the outpatient department register. Simple random sampling was used using the register to identify the study unit with the assumption that one eligible household member to participate, and a non-response rate of 10% (i.e., 1.11 inflation factor). We assumed an intra-cluster correlation coefficient (ICC), a measure of how similar persons from the same health facility are to one another, to be 0.167. Therefore, in the end a total of 1,240 participants required in each group and a total of 2,480 participants overall.

For the baseline survey, most results are reported based on a pooled dataset that includes both planned intervention and control sites (i.e., all 2,480 participants included).

The survey was sampled eligible patients living with hypertension using a random sampling framework. From the existing RTSL support seven regions, two of the regions (Somali and Tigray) were excluded from the study for two reasons. At the time of the survey, the Tigray was a region with security problem with an ongoing active war by the time of the data collection while Somali region has inadequate (less than 30) hypertension patients among five PHC facilities before April 2020. Due to the reasons stated the two regions were excluded from the study. Using a one-to-one ratio of the interventions and control sites 32 Primary Health care Facilities were included in the survey from both groups.

Survey Procedures

Data collection: A structured digital survey was administered by trained healthcare provider data collectors assigned to the participant's woreda using a digital device (smartphone or tablet). Because of sporadic cellular network service coverage, an offline data collection software was used with the plan to download survey responses to a central server as soon as internet service becomes available. Survey questions was administered by trained enumerators hired for this

survey purpose and supervisors checked data quality at a spot and support the enumerators regularly and as needed.

Survey Measures

The baseline Essential Hypertension Services survey solicited identifying and demographic information as well as responses in four domains related to essential hypertension care services.

1. Identifying and demographic information
2. Health services access and financing information
3. Missed or delayed medical services
4. Continuity of HTN services
    - Continuity of blood pressure monitoring
    - Continuity of access to antihypertensive medications

Identifying information was collected at baseline and will be used solely for the purpose of identifying and verifying identity of respondents for the follow-up survey. The survey questionnaire follows this protocol (**Supplementary Appendix B**). Demographic and health services questions asked to all participants and supply information on participant age, sex, educational level, income, woreda of residence, travel time and method of reaching the PHC, and method of financing hypertension care (insurance coverage, fee waivers or out-of-pocket payment for PHC visits and medications). The follow up survey at 12 months will administer the same questions to the same participants surveyed at baseline.

Data Storage and Management

Resolve to Save Lives is a not-for-profit, global public health initiative that is funding and implementing the essential health services project in collaboration with MOH. Resolve to Save Lives has been involved by providing technical support to the survey protocol design from the inception of the idea to drafting as well as pulling together the collaborator. Resolve to Save Lives assigned technical team that is leading the project implementation, PI for the survey and all other logistics including the cost of the survey.

Data were automatically uploaded and stored to a secure, password-protected central server in MoH server. An account was created for this survey data management purpose and only the study investigators have been accessed to the account to do data cleaning and analysis. The enumerators or supervisors only have access to collect and submit data to the server. Individual patient identifiers with not be shared with the project investigators or the data analyzers.

Statistical Analysis

For the baseline survey, control and intervention patient data were pooled and analyzed descriptively including the main dependent variable (i.e., the proportion of hypertensive care service disruption). Descriptive statistics intends to measure frequency of follow-up blood pressure measurements, taking of prescribed medicines, current mode of receiving prescribed medicines, and preferences for receiving medications. Logistic regression analysis was done to determine relationships between the predictor variables and the dependent variable only in intervention sites. This help to identify factors associated with hypertension service disruption and to guide the essential health service packages accordingly. To identify variables that could have association with the dependent variable, first a bivariate logistic regression analysis was used primarily to identify the candidate variables for association with the dependent variable individually. Variables which have p-value less than 0.05 in the bivariate analysis were then entered into multivariable logistic regression for controlling the effects of five possible confounders: survey participant age, sex, income, education level and residence (urban, semi-urban, or rural). Variables with significant association were identified on the basis of adjusted odds ratio (AOR) and p-value <0.05. Moreover, the goodness of fit of the final logistic model was tested using Homers and Lemeshow test at a P-value 0.199 which was considered to be statistically significant.

Ethical Considerations

Primary local ethical approval was obtained from Armauer Hansen Research Institute (AHRI) located in Addis Ababa, Ethiopia. After local approval by the AHRI IRB, due to funding from the U.S.-based non-profit Resolve to Save Lives, the protocol was submitted to the United States-based Biomedical Research Alliance of New York (BRANY) IRB approval. After obtaining the Ethiopia- and US-based IRB committee permissions, the study was conducted by recruiting participants who met the inclusion criteria by filling an informed consent form in their preferred, primary language (usually the local language of the region) containing data about the rights, the role of interviewees, and the expected risks and benefits that might result from participating in the study as a written consent to give their consent. The English language version of this form is available as **Supplementary Appendix C**. Regional language versions of the consent form were translated then back-translated by research staff fluent in all of the relevant languages; these language-specific consent forms were also approved by the AHRI IRB. Each participant was signed the informed consent form in their preferred language before their interview began.

Results

Survey yields

Of 2,480 patients randomly selected from PHC hypertension registers, 2,220 respondents were eligible and were contacted. Of those contacted, 2177(88%) agreed to participate and provided informed consent and were subsequently enrolled in the survey. In the overall sample participation in the planned intervention sites had 53.1% participation (1156 participants) and the control sites had 46.9% participation (1,021 participants). Nearly one-third (32.8%) of the respondents were from Addis Ababa, followed by the Amhara region (24.9%) and Oromia region (18.7%). The actual refusal rate is less than five percent which is 2% (43). The main reason for low response rate is that the survey was interrupted on October 30th, 2021, due to worsening country's security in consultation with Institutional Review Board, and global office.

Discussion

Chronic factors affected hypertension patient retention in hypertension care in Ethiopia before the COVID-19 pandemic. This baseline essential medical services survey documented that barriers to retention in have persisted despite evidence from other sources that Covid-19 has not continued to make a substantial impact on essential medical services in Ethiopia after the initial

Covid-19 response in 2020. This study assessed the magnitude of hypertension care disruption in Ethiopia and identified factors that contribute to missed hypertension management visits.

Magnitude of hypertension care disruption at primary health care units

Around 18% of the hypertension patients participating in this survey had delayed or missed their visit schedule for their routine hypertension care. All of those who missed visits implicated the Covid-19 pandemic as a reason. Other important factors leading to missed visits were need to pay out of pocket for services, including medicines, and living >= one hour travel time from the primary health care facility.

This finding is consistent with an interrupted time series study conducted by other in ten countries found declines in diabetes or hypertension visits of more than 20% in six countries namely Chile, Haiti, Mexico, Nepal, South Africa and Thailand. For example, the time series study conducted in Mexico found that a routine consultation for diabetes and hypertension declined by 32% during the first 9 months of the pandemic [16]. Similarly, a study conducted in India showed that least 50-60% of hypertensive patients do not visit the health facility for their scheduled follow-up appointments [17]. However, despite the reports of hypertension services disruptions reported in our baseline survey, it was not likely that Covid-19 continue to interfere with service delivery. Over time, Ethiopia has been among the least affected countries by health service disruptions during COVID 19 pandemic [15].

The practical and public health implications of our finings is that the high proportion of patients with missed visits challenges the hypertension program in achieving its objectives because continuity of medical treatment is a major factor for achieving population blood pressure control goals. Patients with NCDs require continuous and timely care to prevent complications and improve the quality of care [17], while Primary health care units have an important role to play in ensuring continued access to care, to avoid complications of hypertension and cardiovascular disease [18].

Non-Covid factors associated with hypertension care disruption at selected primary health care units

Three factors: appointment schedule, payment scheme and time to reach to health facility have statistically significant independent factors for hypertension service disruption among primary health care units.

Accordingly, our finding indicated that out-of-pocket payment scheme is one major factor for hypertension patients to miss their appointment. This finding could reflect the issue of financial

risk protection is becoming an emerging national challenge for NCD services and programs as it is clearly indicated in various national documents. For example, according to National Health Accounts 6th report, 68% of NCDI services in Ethiopia were financed by Out of Pocket (OOP) expenditures from household's [21]. Similarly, the national health sector transformation plan II document stated that around 27% of Addis Ababa patients affected with cardiovascular diseases had experienced catastrophic health expenditures, while estimated higher burden in low-income households of patients residing outside of Addis Ababa [22]. Given the general fact that NCDs contributes for the majority of premature death, disability, reduces labor productivity (due to absenteeism), this figure implies the combined huge economic loss of NCDs in terms of labor productivity and health care expenditure in Ethiopia. According to a Nigerian study conducted in 2014, the financial constraints showed an adverse impact on medication adherence, leading to the sub-optimal achievement of BP control [23]. Hence, in line with the concept of "universal health coverage", national NCDs programs, might need to redesign the current model of care or interventions aimed to reduce financial barrier for NCD patients and cardiovascular diseases in particular.

Besides, our finding indicated that longer time to reach to health facility (i.e. > one hour) was also found to be an important factors leading to missed appointments. Although we assessed distance to reach health facility by time, our findings correlate with the study finding conducted in Addis Ababa Ethiopia, where 22.3% of respondents have raised distance between patient's residence and the facility as a reason for missing their Diabetes or hypertension care visit [19]. Regarding the appointment schedule system, there are evidences service promoted service continuity during the pandemic, including scheduling visits to reduce overcrowding, community delivery of drugs for patients with stable chronic conditions, weekend opening hours and special vaccination campaigns or health days [15] .For example, Differentiated Service Delivery (DSD) model were found to be the contemporary patient centered service delivery model being proven on ART program across various countries, where a study conducted across four countries showed that the number of people on ART was virtually unaffected during the pandemic[15]. Similarly, there is evidence from India where decentralized, patient-centered care were used as mitigation strategy to ensure the continuation of essential health services for patients with chronic diseases [24]. While some studies recommend patients to have the ability to obtain a 90-day supply of medication to reduce likelihood of missed doses [18].

Hence, in the light of the increasing burden of NCDs on health systems in low- and middle-income countries, particularly in Sub-Saharan Africa, context-adapted, cost-effective hypertension

service delivery models based on the WHO-HEARTS technical package are now urgently needed [25].

Limitations and Challenges

Because selection of intervention facilities was performed independently of the survey and is not a random sample, population characteristics might mean that participants who seek care at intervention facilities are systematically different than persons who seek care in control facilities. However, matching was done in selecting the woredas and health facilities participated in the study using the geographic, sociocultural and health facilities national standard of classification. This still can result in a bias in sample estimates. Intervention facilities were also implementing hypertension program components that may affect survey responses along pathways unrelated to the Essential Services program which is difficult to measure. Lastly, the background activity of COVID-19 and government responses to the epidemic vary by region and woreda as well as disrupt the Essential Services Project and community survey. This could lead to confounding in ways that are impossible to anticipate.

In addition, because of the country security situation by the time of data collection, the researcher team forced to suspended data collection before reaching the targeted sample size particularly in two regions (Amhara and Oromia region). However, this study data collection able to complete 88% with greater than 96% response. The termination of the data collection was decided with consultation and notification of the IRB institutes and senior global co investigators.

Conclusions and Recommendations

Conclusion

This study highlighted the challenges related to access; the travel time to reach the health facility, in which the study revealed it is a barrier for continuous follow up visit, hypertension service provision such as waiting time and appointment spacing which indicated the importance of patient center approach. Moreover, the study revealed that system and economic factors have a role in hypertension service disruption. More than one sixth (18%) of the study participants delayed or missed their schedule visit and service payment modality and the economic impact of COVID-19 pandemic has significant role for patients to miss their visit.

Recommendation

Based on the study finding we recommend

- The survey findings indicated financial protection for is very important to ensure equitable hypertension service uptake and retention in care. Ethiopia should strengthen the expansion of community-based health insurance to promote service and ensure continuity of care as well as patient retention in care. Moreover, to address the economic factors that challenges patients to afford care service and medication cost, the government and partners should work on policy or a strategy that helps patients to access affordable care services and medication in the possible least cost or free medication.
- To minimize missed visit by the patients, a patient-centered service delivery modality should be implemented. The mode should base on the accessibility of the services through provision of drug refill services and perhaps blood pressure monitoring at the community level.
- Building resilient Primary health care services that copes with challenging situations such as COVID-19 is necessary to ensure maintenance of continuous essential hypertension services. All stakeholders must join the effort build the capacity of the primary health care and community response structures to weather the inevitable storms that will threaten do disrupt hypertension care.

References

1. World Health Organization/WHO (2021) Factsheet Hypertension, available at https://www.who.int/news-room/fact-sheets/detail/hypertension
2. K. Juma, P. A. Juma, C. Shumba et al., "non-communicable diseases and urbanization in African cities: a narrative review," in Non-communicable Diseases and Urbanization-A Global PerspectiveIntechOpen, London, UK, 2019.
3. V. Mwenda, M. Mwangi, L. Nyanjau et al., "Dietary risk factors for non-communicable diseases in Kenya: findings of the STEPS survey, 2015," BMC Public Health, vol. 18, no. 3, p. 1218, 2018.
4. B. Twinamasiko, E. Lukenge, S. Nabawanga et al., "Sedentary lifestyle and hypertension in a peri-urban area of Mbarara, South western Uganda: a population based cross sectional survey," International Journal of Hypertension, vol. 2018, Article ID 8253948, 8 pages, 2018.

5. Kibret KT, Mesfin YM. (2015) Prevalence of hypertension in Ethiopia: A systematic meta-analysis, Public Health
6. Horsa & Tadesse (2019). Assessment of hypertension control and factors associated with the control among hypertensive patients attending at Zewditu Memorial Hospital: a cross sectional stud, BMC Research Notes
7. Ministry of Health/MoH (2019) Essential Health Services Package of Ethiopia available at https://www.humanitarianresponse.info/en/operations/ethiopia/document/essential-health-services-package-ethiopia-2019
8. Fiscella K, Holt K. Racial disparity in hypertension control: tallying the death toll. Ann Fam Med. 2008;6:497–502.
9. Onwukwe SC, Omole OB. Drug therapy, lifestyle modifcation and blood pressure control in a primary care facility, south of Johannesburg, South Africa: an audit of hypertension management. S Afr Fam Pract. 2012;54(2):156–61.
10. Chobanian AV, Bakris GL, Black HR, Cushman WC, Green LA, Izzo JL, et al. The seventh report of the Joint National Committee on Prevention, Detection, Evaluation, and Treatment of High Blood Pressure: the JNC 7 Report. JAMA. 2003;289:2560–72.
11. Mancia G, Fagard R, Narkiewicz K, Redo´n J, Zanchetti A, Bo¨hm M, et al. ESH/ESC Guidelines for the management of arterial hypertension: the Task Force for the management of arterial hypertension of the European Society of Hypertension (ESH) and of the European Society of Cardiology (ESC). J Hypertens. 2013;31:1281–357.
12. Goverwa TP, Masuka N, Tshimanga M, Gombe NT, Takundwa L, Bangure D, et al. Uncontrolled hypertension among hypertensive patients on treatment in Lupane District, Zimbabwe, 2012. BMC Res Notes. 2014;7:703.
13. World Health Organization/WHO (2021) available Coronavirus disease (COVID-19) (who.int)
14. Partnership for Evidence-based Response to COVID-19 (2020) Available at PERC | Prevent Epidemics.
15. Catherine A. et.al. COVID-19 and resilience of healthcare systems in ten countries. Nature Medicine. 2021. https://doi.org/10.1038/s41591-022-01750-1
16. Doubova SV et al. Disruption in essential health services in Mexico during COVID-19: an
17. interrupted time series analysis of health information system data. BMJ Global Health 2021;6:e006204. doi:10.1136/bmjgh-2021-006204
18. Das B, Neupane D, Singh Gill S, Bir Singh G. Factors affecting non-adherence to medical appointments among patients with hypertension at public health facilities in Punjab, India. J Clin Hypertens. 2021;23:713–719. https://doi.org/10.1111/jch.14142
19. PAHO. Managing people with Hypertension and Cardio vascular Disease during COVID-19. Pan American Health Organization, WHO. 2020.
20. Shimels T, Asrat Kassu R, Bogale G, Bekele M, Getnet M, Getachew A, et al. (2021) Magnitude and associated factors of poor medication adherence among diabetic and hypertensive patients visiting public health facilities in Ethiopia during the COVID-19 pandemic. PLoS ONE 16(4): e0249222. https://doi.org/10.1371/journal.pone.0249222
21. Federal Ministry of Health, Ethiopia/MOH (2020) Health sector monthly analytic report, Policy Plan Monitoring and Evaluation Directorate
22. Ministry of Health-Ethiopia. NATIONAL STRATEGIC PLAN FOR THE PREVENTION AND CONTROL OF MAJOR NON-COMMUNICABLE DISEASES (2020/21-2024/25). July 2020

23. Ministry of Health-Ethiopia, Health sector Transformation Plan II, HSTP II 2020/21-2024/25, Feb. 2021
24. Okwuonu et al. Patient-related barriers to hypertension control in a Nigerian population. International Journal of General Medicine. Dove Press. 2014:7 345–353
25. Kunwar A, et al. Interventions to Ensure the Continuum of Care for Hypertension During the COVID-19 Pandemic in Five Indian States—India Hypertension Control Initiative. Global Heart. 2021; 16(1): 82. DOI: https://doi.org/10.5334/gh.1010
26. Frieden et al. Setting up a nurse-led model of care for management of hypertension and diabetes mellitus in a high HIV prevalence context in rural Zimbabwe: a descriptive study BMC Health Services Research (2020) 20:486 https://doi.org/10.1186/s12913-020-05351
