# Supplementary material for: Factors Associated with Hypertension Care Follow-Up in the Ethiopia HEARTS Program
Source: Glob Heart. 2025 Feb 26;20(1):20. doi: 10.5334/gh.1407 (PMC11869820; doi:10.5334/gh.1407)
Supplement: Supplementary Material. — Appendix B. [file gh-20-1-1407-s2.pdf]

Annex

1. Baseline Survey Questionnaire

I. *Demographics*

Question 1: **Age?** ________ (years)

Question 2: **Gender?**

1. Male
2. Female

Question 3: **What is your residence address?**

1. Region: ______________
2. Woreda/Town: ______________
3. Kebele: ______________

Question 4: **Type of Residence:**

1. Urban
2. Rural
3. Semi Urban

Question 5: **Annual income? ___________ *birr***

If you don't know the exact amount, please choose from the range below

1. Less than 12,000 Birr
2. 12,000 ≤ 18,000 Birr
3. More than 18,000 ≤23,300
4. More than 23,300 ≤ 30,000
5. More than 30,000
6. Don't Know
7. Refused

Question 6: what is your educational status?

1. No formal schooling
2. Less than primary school
3. Primary school completed
4. Secondary school completed
5. Tertiary (College/University completed and or post graduate degree)
6. Refused

II. Health Services

Question 7: **Means of transportation for reaching the PHC?**

1. Public Transportation
2. Private car
3. Walking
4. Bicycle
5. Motorcar/Bajaje or motorcycle
6. Horse cart
7. Combination of the above
8. Others ________________

Question 8: **How long does it take you to reach the PHC?**

1. ≤10 minutes
2. 11 to 30 minutes
3. 31 to 60 minutes
4. >60 minutes

Question 9: **How long does it take you to get the services starting from registering to be seen by the HCW at each visit?**

1. <1 hours
2. 1 to 3 hours
3. Half days
4. Full days

Question 10: **How do you pay for your HTN care?**

1. Insurance coverage
2. Out-of-Pocket
3. Free waiver
4. Other (specify): __________________

III. Missed or delayed essential medical services

Question 11: **how often your appointment is assigned for the next visit?**

1. Every 28 days
2. Every two month
3. Every three month
4. Currently, discontinue follow up
5. Other

Question 12: **Have you delayed, skipped or /missed follow-up in the past six months? If NO, skip to Question 14a. If YES, go to Question 13.**

1. Yes
2. No

Question 13: **Why you have delayed, skipped or been unable to complete health care visits in the past six months (may choose more than one response)?**

1. Covid Related:
   1.1. Fear of contracting COVID-19 at the health facility
   1.2. Could not afford services due to COVID-19 impact on my income
   1.3. Health facilities were closed due to COVID-19
   1.4. I was unaware the health facilities were open for essential services during COVID-19
2. Other, non-Covid related reason:
   2.1 Could not afford services
   2.2 Medication cost
   2.3 Health facilities were too busy/had limited staff available
   2.4 Medications were not available when I went to the facility
   2.5 I did not feel sick/I feel healthy
   2.6 Went to holy water/traditional healer & I have been monitoring myself with no issues
   2.7 Other

IV. Continuity of blood pressure monitoring

Question 14a: **When was the last time you had your blood pressure measured (choose one)?**

1. Within the past week
2. More than one week ago, but within the prior month
3. Between 1-3 months ago
4. More than 3 months ago
5. Can't recall when I received last BP measurement
6. Other

Question 14b: **Where did you last get your blood pressure measured (may choose more than one)?**

1. Hospital
2. Health center (in the catchment are health center/the study unit)
3. Health post
4. Private clinics
5. At homes by HEWs or family member
6. Can't recall last BP measurement
7. Other (specify): ________________

V. *Continuity of access to antihypertensive medications and mode of antihypertensive medication delivery*

Question 15a: **When was the last time you took your prescribed HTN medication(s)?**

(choose only one)

1. I took my daily medicine(s) today/last night
2. Not today, but within the past week
3. Not this week, but within the prior month
4. 1-3 months ago
5. More than 3 months ago
6. Discontinue medication
7. I am not prescribed medicine(s)
8. Other

Question 15b: **Have you had any difficulty to take or to get your prescribed medications?**

1. Yes
2. No
3. Not applicable (for patients LSM)

If NO, skip to Question 15e. and skip if N/A to Question 16.

Question 15c: **Why do you have difficulty taking/get your prescribed medications? (Mark all that apply)**

1. The medicines don't help me feel better, so I don't take them
2. I experienced side effect symptoms, so I stopped taking the medicine
3. I want to take my medicines, but i can't remember to take my medicines every day
4. I am busy and it is too challenging to take daily medicines
5. I cannot afford to pay for my daily medicines
6. Medication is not available
7. Other

Question 15d: **Due to difficulty obtaining your prescribed medications, how many doses you skipped taking medications? (This question will be Applicable only If they are taking the medicine from the study Health center)**

1. I missed all doses
2. I skipped one or more doses per week
3. I skipped doses rarely, but I take it daily in most weeks
4. Discontinue
5. Other Specify

Question 15e: **How do you currently receive your regular medicine(s)? (May choose more than one)**

1. From a hospital/health center pharmacy
2. From government pharmacy outside of the health facility
3. From a local, privately owned pharmacy/drug store
4. From abroad (through relatives or other)
5. Some combination of above options

6. Other specify

Question 15f: **How would you prefer to receive your regular medicine(s)?**

1. From a hospital/Health center pharmacy
2. From government pharmacy outside of the health facility
3. From a local, privately owned pharmacy
4. Community delivery (homes delivery or delivery to post)
5. Other specify

Question 16: **How frequent do you want to visit the health facility and get medication?**

1. From Health facility every month
2. From health facility every two months
3. From health facility every three months
4. From health facility every six months
5. Other specify

Question 17: **during your follow up visit, who usually provide your clinical services and drug refill prescriptions?**

1. Physicians
2. Health Officer
3. Nurses
4. I don't know
5. Other specify

2. Study Intervention and Control Sites

Region	Name of Health care facility	Type of Health care facility	Study Group
Oromia	1. Awash	Health care center	Control
	2. Adama	Health care center	Control
	3. Biyo	Health care center	Intervention
	4. Doni	Health care center	Intervention
	5. Dire	Health care center	Control
	6. Dhankaka	Health care center	Control
	7. Ejere	Health care center	Intervention
	8. Gefersa	Health care center	Control
	9. Gode dera	Health care center	Intervention
	10. Geda	Health care center	Control
	11. Galdia	Health care center	Control
	12. Hachaltu	Health care center	Intervention
	13. Kuriftu	Health care center	Control
	14. Koka	Health care center	Intervention

	15. Mojo	Health care center	Intervention
	16. Olinchiti	Health care center	Intervention
	17. Olinchiti	Hospital	Intervention
	18. Shewa	Health care center	Control
	19. Tade	Health care center	Intervention
	20. Wonji	Hospital	Control
Amhara	21. Addis Alem	Hospital	Intervention
	22. Bahir Dar	Health care center	Control
	23. Birakate	Health care center	Intervention
	24. Han	Health care center	Control
	25. Merawi HC	Health care center	Intervention
	26. Merawi Hospital	Hospital	Intervention
	27. Meshenti	Health care center	Control
	28. RIM	Health care center	Intervention
	29. Shumabo	Health care center	Intervention
	30. Tagel	Health care center	Intervention
	31. Tis Abay	Health care center	Control
	32. Zegie	Health care center	Control
	33. Shimbit	Health care center	Control
	34. Abay	Health care center	Control
Addis Ababa	35. Addis Hiwot	Health care center	Intervention
	36. Addisu Gebeya	Health care center	Control
	37. Afincho Ber	Health care center	Control
	38. Arada	Health care center	Control
	39. Churchil	Health care center	Control
	40. Entoto Fana	Health care center	Intervention
	41. Hidase	Health care center	Intervention
	42. Janmeda	Health care center	Control
	43. Kebena	Health care center	Control
	44. Maychew	Health care center	Intervention
	45. Ras Emiru	Health care center	Control
	46. Semen	Health care center	Control
	47. Shiro Meda	Health care center	Intervention
	48. St. Peter	Hospital	Intervention
	49. Tibeb Bekechene	Health care center	Intervention
	50. Shegole	Health care center	Intervention
Dire Dawa	51. Dechatu	Health care center	Intervention
	52. Dire Dawa	Health care center	Control
	53. Dilchora	Health care center	Control
	54. Goro	Health care center	Intervention
	55. Genda Kore	Health care center	Control
	56. Genda Gerada	Health care center	Intervention

	57. Melka Jebdu	Health care center	Intervention
	58. Sabian	Hospital	Intervention
	59. A/Ketema	Health care center	Control
	60. Lege Hare	Health care center	Control
Sidama	61. Yaye	Hospital	Intervention
	62. Daye	Hospital	Intervention
	63. Yibra	Hospital	Control
	64. Tulla	Hospital	Control
