# Supplementary material for: Factors Associated with Hypertension Care Follow-Up in the Ethiopia HEARTS Program
Source: Glob Heart. 2025 Feb 26;20(1):20. doi: 10.5334/gh.1407 (PMC11869820; doi:10.5334/gh.1407)
Supplement: Supplementary Material. — Appendix C. [file gh-20-1-1407-s3.pdf]

Supplementary Appendix C

Informed Consent Form (English language version)

Informed Consent

All participants meeting inclusion criteria will be informed about the benefits and risks of participating in this survey in their preferred language and all participants provided written informed consent prior to survey enrollment. This protocol was approved by the Amrauer Hansen Research Institute (AHRI).

Text to be read to potential participants prior to the baseline survey:

Hello. My name is {Data Collector's Name }. I am working with a public health organization called Resolve to Save Lives (RTSL). We are conducting a survey about the state of essential medical services in Ethiopian communities during the time of the Covid-19 epidemic. The information we collect will help RTSL and the national and regional governments to evaluate the state of essential medical services for people who are treated for high blood pressure in primary health care centres. You have been informed about this survey previously by _______________ (Name of the CHW assigned to the person's area)

"We are inviting you to participate in the Ethiopia essential medical services survey. If you agree to participate, you will answer the following questions about your basic personal information as well as your use of general health services and hypertension health services over the last 6 months. Within the next year, we will return to ask you the same set of questions. This information will be kept confidential and will not be shared with anyone. This survey has been approved by an ethics review committee at the the Amrauer Hansen Research Institute (AHRI) in Addis. Your decision to take part in the survey or refusal to participate will not affect the care that you will be receiving from your primary health care facility. Participation is this study merely depends on your free will."

Introduction and Purpose of the Study: The Ethiopia Essential Services Project is working to improve access to essential medical care for people like you. This survey will provide important information about your use of medical services.

Description of the Research: When you agree to this study, you will be asked to answer a series of questions about your use of standard medical services.

Subject Participation: Participation in this survey is voluntary. You can exit the survey at any time. You can also decline to participate in the planned follow up survey.

Potential Risks and Discomforts: This survey consists of a series of questions only. No questions will pertain to the state of your health or any other sensitive personal information.

Potential Benefits: This survey will not likely benefit you directly. However, the information learned in this survey may improve medical care for Ethiopians in general.

Thank you for agreeing to learn about this survey.
